# Supplementary material for: The Construction and Exploration of a Comprehensive MicroRNA Centered Regulatory Network in Foxtail Millet (Setaria italica L.)
Source: Front Plant Sci. 2022 May 6;13:848474. doi: 10.3389/fpls.2022.848474 (PMC9121102; doi:10.3389/fpls.2022.848474)
Supplement: Supplementary file 3 [file Data_Sheet_3.zip › Sit-miR408_Seita.7G064700.1_153_TPlot.pdf]

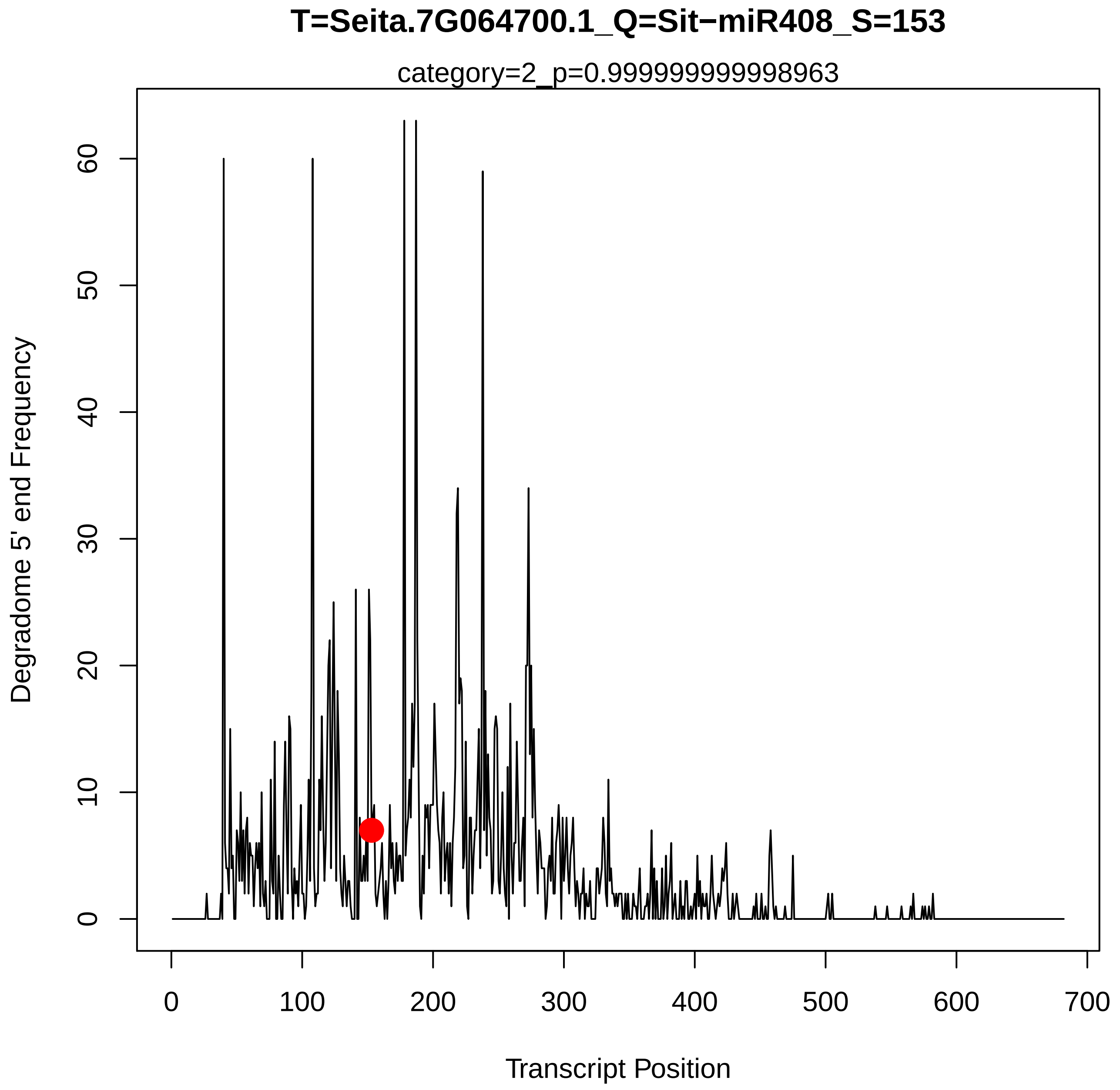
T=Seita.7G064700.1_Q=Sit−miR408_S=153
category=2_p=0.999999999998963
Degradome 5' end Frequency
0
10
20
30
40
50
60
0
100
200
300
400
500
600
700
Transcript Position
